# Cervical verrucous cancer misdiagnosed with verrucous hyperplasia: A case report

**DOI:** 10.1016/j.heliyon.2022.e10268

**Published:** 2022-08-17

**Authors:** Yi-Ting Hsu, Kim-Seng Law

**Affiliations:** a Department of Obstetrics and Gynecology, Tung's Taichung MetroHarbor Hospital, Taichung, Taiwan; b Department of Obstetrics and Gynecology, Tung's Taichung MetroHarbor Hospital, Taichung, Taiwan; c Department of Nursing, Jenteh Junior College of Medicine, Nursing and Management, Miaoli, Taiwan; d Department of Post-Baccalaureate Medicine, College of Medicine, National Chung Hsing University, Taichung, Taiwan

**Keywords:** Verrucous carcinoma, Cervical neoplasm, Superficial biopsy, Lymph node metastasis

## Abstract

**Background:**

There are many different variants of squamous cell carcinoma (SCC), and verrucous carcinoma (VC) is a rare and highly differentiated SCC. Due to its preference of local invasion, regional lymphatic involvement rarely occurs. VC is difficult to diagnose using conventional pap smear or cervical punch biopsy, in which adequate stroma including bulbous rete pegs is required for a definitive diagnosis. Surgical management is recommended as the first-line treatment with radiotherapy forbidden due to the risk of anaplastic transformation.

**Case report:**

We presented a 59-year-old Taiwanese female who had postmenopausal bleeding for three months with two consecutive normal pap smear and biopsy at other hospital. Pelvic examination showed a necrotic fungating cervical mass with upper 1/3 vaginal involvement. Colposcopic guided cervical biopsy and fractional dilatation and curettage revealed verrucous hyperplasia (VH) with negative high-risk HPV typing. Pelvic 3T magnetic resonance imaging (MRI) was arranged, and a 3.7 × 3.6 × 4.0 cm necrotic mass at the cervix with an enlarged left pelvic lymph node was found. Positron emission tomography with computed tomography (PET/CT) demonstrated avid uptake at the cervix and left pelvic lymph node. Surgical intervention was performed due to highly suspicious of cervical verrucous carcinoma with positive pelvic lymph node. The final pathologic report was a well-differentiated verrucous carcinoma, IIA2 by International Federation of Gynecology and Obstetrics (FIGO) classification.

**Conclusion:**

VC is difficult to diagnose preoperatively, and surgical excision is recommended as the first-line treatment.

## 1 Introduction

Verrucous carcinoma (VC) is a rare variant of highly differentiated squamous cell carcinoma (SCC), and the first case was reported in 1948 [1]. Although oral cavity is the most common site, it may also develop in the female genital tract, including the cervix, vulva and vagina [2, 3]. Previous research demonstrated the association between human papilloma virus (HPV) infection and this particular type of cancer [4]. Compared with females of childbearing age, those in ilate reproductive age and postmenopausal period are more vulnerable to VC. Due to its slow-growing and locally invasive characteristics, regional lymphatic involvement is rare, and distant metastasis is even scarcer [5].

It is difficult to differentiate between VC, VH and invasive SCC simply by punch biopsy. Definite diagnosis often needs a more extensive biopsy, which may demonstrated the pattern of rete ridge at the base of the tumor [6]. With regard to treatment of VC, surgical excision is recommended as first-line management [7]. Radiotherapy is not recommended due to the risk of iatrogenic anaplastic transformation as well as further regional and distant metastasis [8]. In respect to the paucity of reported cervical VC cases, our study aimed to report a rare case with diagnostic challenges.

## 2 Case report

A 59-year-old Taiwanese woman with an unremarkable medical history visited our outpatient department due to post-menopausal bleeding and back soreness for three months on April 2020. The report of her previous Papanicolaou smears and cervical biopsy was normal. Pelvic examination found a necrotic cervical mass with upper third vaginal involvement, and multiple cervical biopsies revealed VH. Informed consent for data publication was collected from the patient.

**Figure 1:**
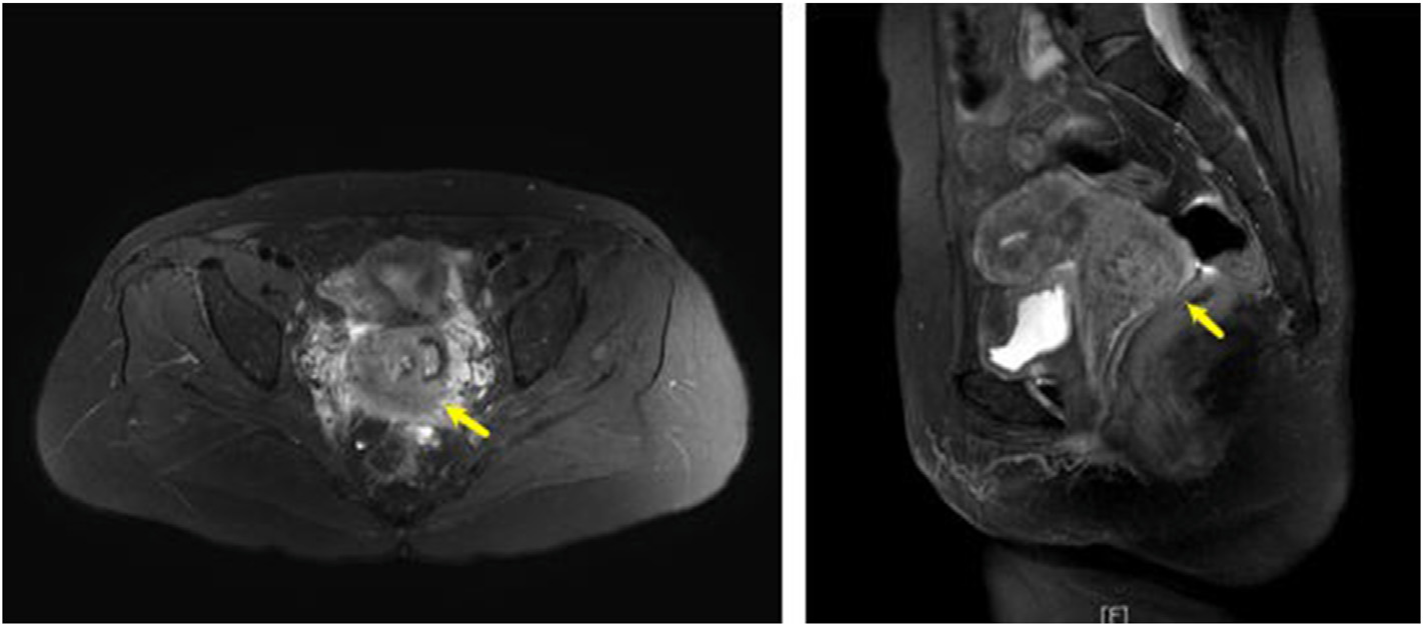
The pelvic 3T MRI showed a 3.7 × 3.6 × 4.0 cm necrotic mass with internal heterogeneous signals on T2-weighted image. (a) Axial view (b) Sagittal view.

The pelvic 3T magnetic resonance imaging (MRI) showed a 3.7 × 3.6 × 4.0 cm necrotic mass with peripheral enhancement in the right lateral aspect of the uterine cervix. An enlarged lymph node measuring 1.8cm in diameter was found in the left pelvic cavity posterior to the left external iliac artery (Figure 1).

**Figure 2:**
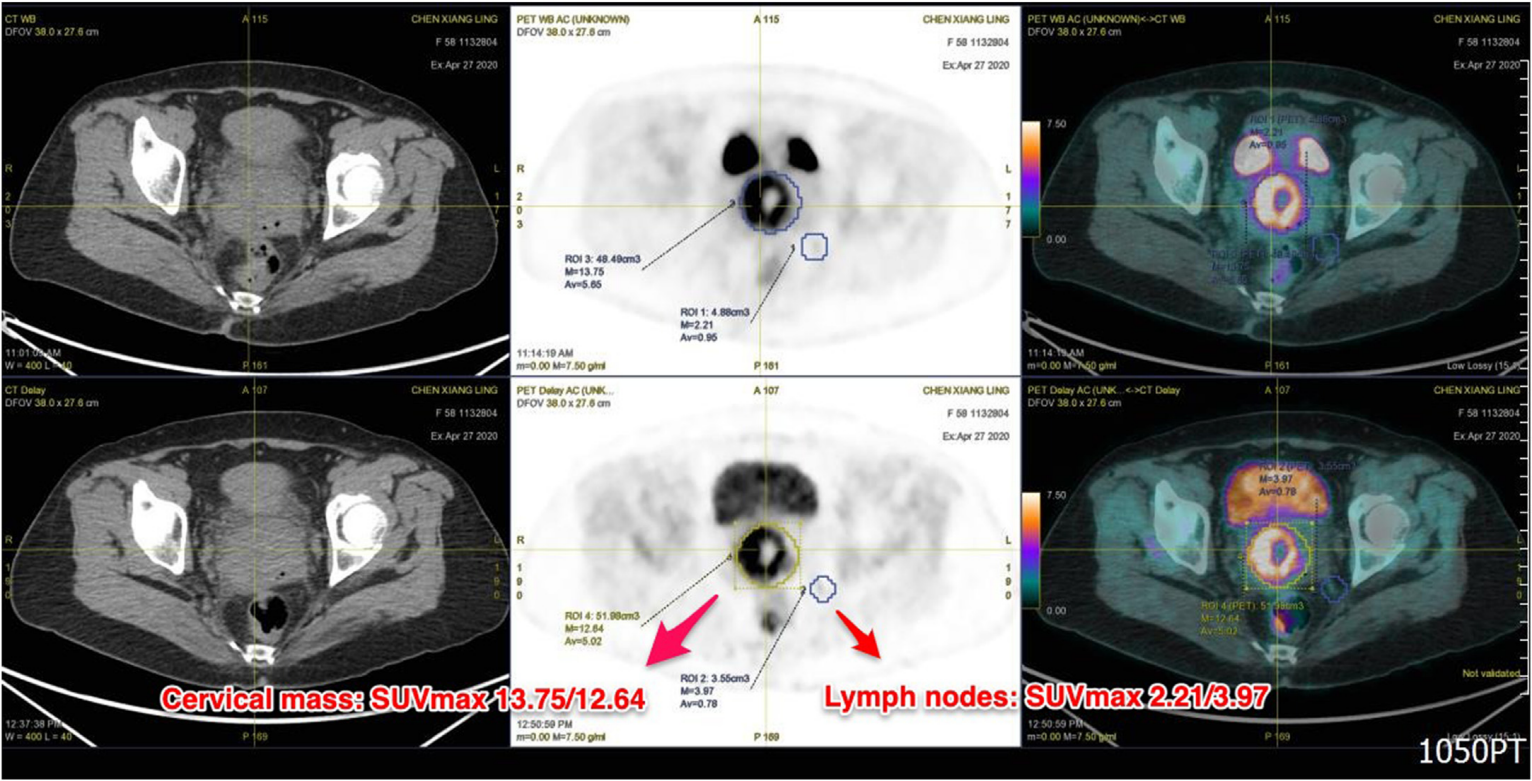
PET/CT with FDG revealed an FDG-avid cervical mass, with invasion of two lymph nodes at the left pelvic cavity.

Positron emission tomography with computed tomography (PET/CT) with F-18-fluorodeoxyglucose (FDG) was performed and demonstrated an FDG-avid cervical mass with intense FDG uptake. The initial and delayed maximum standardized uptake value (SUV_max_) were 13.75 and 12.64, respectively. Besides, there were two FDG-avid lymph nodes at the left pelvic cavity. One measuring 0.94cm in size was at the medial side of the left internal iliac artery, showing increased FDG uptake with initial/delayed SUV_max_ of 2.21/3.97. The other one measuring 1.8cm in diameter posterior to the left external iliac artery, showing moderate FDG uptake with initial/delayed SUV_max_ of 6.84/6.23 respectively (Figure 2). They were compatible with the same lesion under MRI imaging. According to the radiographic findings, cervical malignant tumor was highly suspected.

Due to the discrepancy between the clinico-radiological features and biopsy reports, we performed fractional dilatation and curettage (D&C) for further pathological confirmation prior to surgical intervention. The pathological reports of fractional D&C revealed parakeratosis and VH in the squamous epithelium. Due to the bulky tumor and close approximation to the bladder, a cystoscopy during operation was done with unremarkable finding. Although the pathological report was inconclusive, malignancy was still highly suspected with regard to the clinical and radiological manifestations.

**Figure 3:**
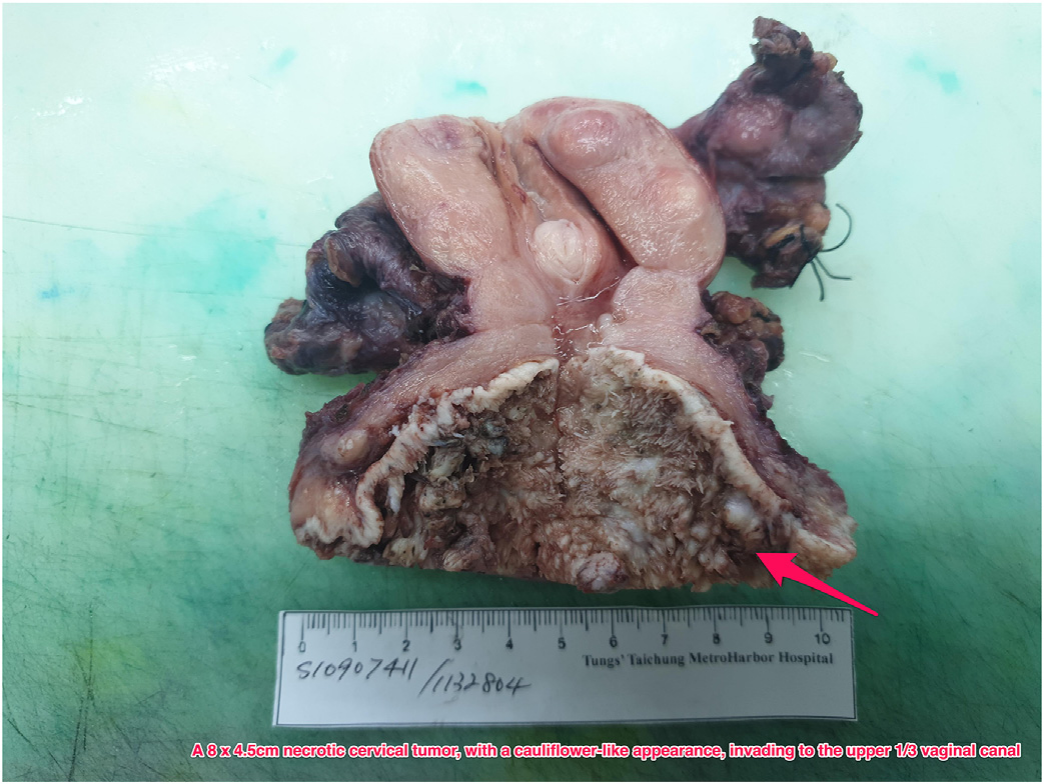
Large exophytic warty mass of the cervix measuring around 8 × 4.5 cm.

Under high suspicion of cervical VC, stage IIIC1r based on the International Federation of Gynecology and Obstetrics (FIGO) staging system, frozen section over left pelvic lymph node was removed first which reveal only lymphoid hyperplasia. We then performed radical hysterectomy with bilateral salpingo-oophorectomy, as well as bilateral pelvic lymph node dissection and partial vaginectomy [9]. An 8 × 4.5cm necrotic cervical tumor, with cauliflower-like appearance, invading to the upper 1/3 vaginal canal was found (Figure 3).

**Figure 4:**
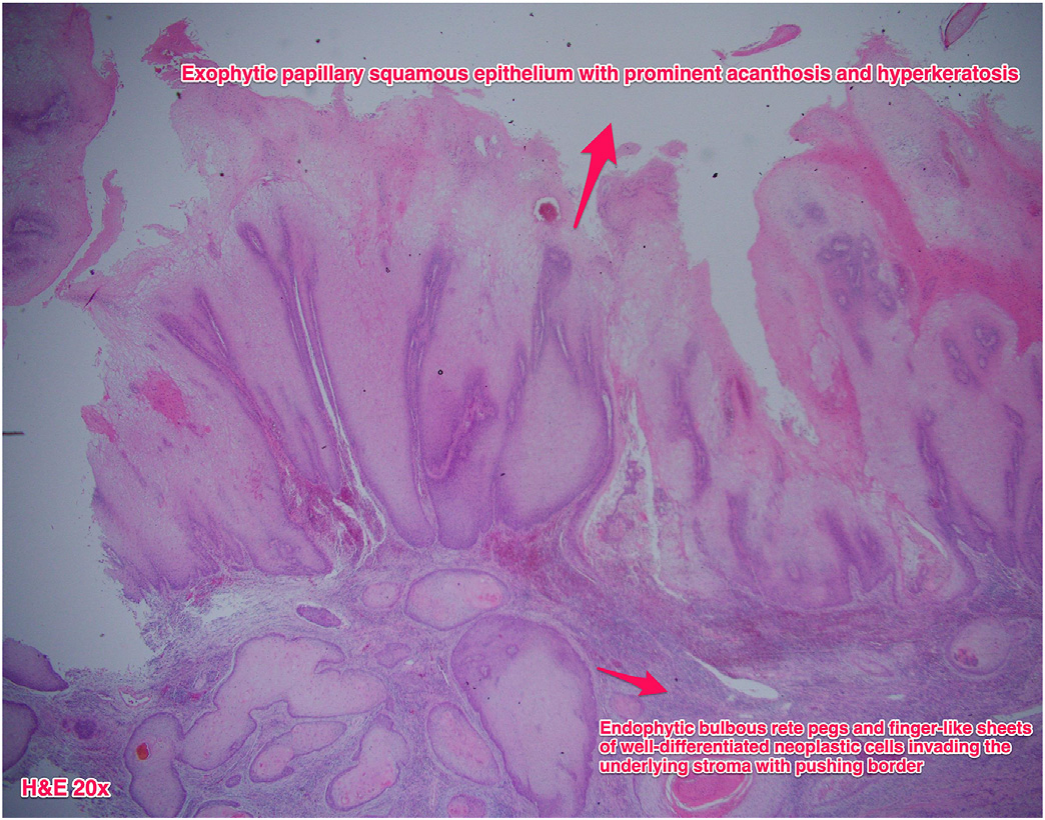
The image showed bulbous and finger-like sheets of well-differentiated neoplastic cells invading the underlying stroma with push border (H & E x 20).

The microscopic findings of the surgical specimen illustrated an exophytic papillary squamous epithelium with apparent acanthosis and hyperkeratosis. There were also endophytic bulbous rete pegs and finger-like sheets of well-differentiated neoplastic cells invading the underlying stroma with pushing border (Figure 4). The parametrium was not involved. The pathological report of the left pelvic lymph nodes only showed reactive hyperplasia. Immunohistochemical staining of HPV was negative. P16 and p53 immunostaining was not over expressed, and only revealed a 10 percent focal positivity. According to the aforementioned histological results, a well-differentiated verrucous carcinoma, IIA2 by FIGO classification, was the final diagnosis [9].

Two months after the surgery, the patient received follow-up at the gynecological outpatient unit. Both the reports of Papanicolaou smear and HPV testing were again normal at the initial follow up. Patient did not receive any chemotherapy or radiotherapy after surgery. Until now, twoyear after the initial therapy, there has been no evidence of recurrence or disease related complications.

## 3 Discussion

VC of the cervix is an extremely rare disease with first case published in 1972 [7]. It displays slow-growing characteristics, and usually presents as a locally aggressive malignant tumor with rare regional or distant metastasis. Although the majority of cases were reported in postmenopausal females, young childbearing individuals were also presented in the literatures [10].

It has distinctive pathologic characteristics. There are multiple exophytic papillary fronds covered with hyperkeratotic cells with endophytic pushing borders invading the underlying stroma via broad bulbous rete pegs of neoplastic cells [2, 7]. It is crucial to distinguish VC from condylomata acuminatum because of their similar gross appearance. The main pathological difference between condylomata acuminatum and VC is the presence of central fibrovascular cores in the epithelial papillae in the former lesion [11].

Besides, as the previous report mentioned, it was difficult to differentiate VH and VC with punch biopsy alone [6]. However, Kallarakkal provided some pathological characteristics to distinguish these two diseases. VH was recognized only by its exophytic growth pattern, whereas, VC had combined exophytic and endophytic growth pattern. With regard to the depth of lesion invasion, VC always showed projections of neoplastic epithelium deep into the adjacent naïve epithelium. This feature was not seen in VH, whose neoplastic epithelium was limited at the same level as the adjacent epithelium [12]. Therefore, sufficient stroma depth including the bulbous rete pegs at the base of the lesion is required for definitive diagnosis, and we suggest extensive cervical biopsy as an initial and major step to diagnose VC. However, a biopsy with such deep stromal sampling is challenging in clinical practice. Thus, in those who are difficult to be diagnosed by cervical biopsy, we recommend PET/CT to differentiate a benign frommalignant tumor with high SUV uptake usually seen in the latter category. If PET/CT is not available, CT or MRI can be used as an alternative for identifying lymph nodes metastasis with limited value of differentiate benign from malignant.

In our case, VH was revealed through cervical biopsy and D&C first. However, after hysterectomy, the final pathological report confirmed the diagnosis of VC. Due to the aforementioned limitation of pathological diagnosis of VH and VC, we recommended further comprehensive radiological assessments, including abdominal CT or MRI and PET, before the diagnosis of VH is achieved. In case of highly suspicious of malignancy, hysterectomy should be planned for definitive diagnosis. To avoid misdiagnosis, the diagnosis of VH should be established only when the susceptibility of VC is fully excluded by the above imaging investigation.

We searched the PubMed database before September 23, 2021 to identify English articles reporting cervical VC. We only accounted for the articles whose full texts were available. Cases which used term of “squamous papillomas” were not mentioned here, because it presented as an outdated term and there was obscure definition between squamous papillomas and VC. A total of 27 cases published from 1972 to 2017 were found (Table 1).

**Table 1:** Summary of the clinical characteristics of 27 cervical VC cases.

Author	Published year	Case(s)	Symptoms	HPV infection	Staging	Primary treatment	Adjuvant treatment	Lymph node metastasis	Follow up time (months)	Outcome
Jennings, R. H. [7]	1972	1	Vaginal bleeding	NA	IB	Trachelectomy∗	nil	Not involved	21	Multiple site recurrences during 7 years
Demian, S. D [8]	1973	1	NA	NA	NA	Radiation therapy	nil	NA	NA	Recurrence, expired during subsequent surgery
Qizilbash, A. H. [15]	1974	2	NA	NA	IB (clinical)	Hysterectomy	nil	NA	60	No signs of recurrence
			NA	NA	IB	Radiation	nil	NA	36	No signs of recurrence
Isaacs, J. H. [16]	1976	3	NA	NA	NA	Radical hysterectomy and pelvic lymph node dissection	nil	NA	36	No signs of recurrence
			NA	NA	IIA	Radical hysterectomy, pelvic lymph node dissection and partial vaginectomy:	nil	NA	84	No signs of recurrence
			NA	NA	IIA	Hysterectomy and partial vaginectomy,	nil	NA	14	No signs of recurrence
Spratt, D. W. [17]	1977	1	Irregular bleeding and abdominal pain	NA	IIA	Radiation therapy	nil	NA	12	No signs of recurrence
Powell, J. L. [18]	1978	1	Bloody vaginal discharge for 2 months	NA	#171717; IVA	#171717; Radiation therapy	Surgical excision, pelvic and periaortic lymphadenectomy	Not involved	11	No signs of recurrence
Rorat, E. [19]	1978	1	Irregular vaginal bleeding	NA	IIA (clinical)	Radical hysterectomy, upper-third vaginectomy, left salpingectomy, bilateral oophorectomy, bilateral pelvic lymphadenectomy, and periaortic LN dissection	nil	NA	12	Recurrence, expired one year after the original diagnosis
Faaborg, L. L. [20]	1979	1	Postmenopausal bleeding	NA	IIA	Radical hysterectomy, pelvic lymphadenectomy, and total vaginectomy	nil	Not involved	7	Recurrent at vagina
Väyrynen, M. [21]	1981	1	Bloody vaginal discharge for 1 week	NA	II (clinical)	Intracavitary radium application	Radical operation	Not involved	>60	No signs of recurrence
Tiltman, A. J. [22]	1982	1	Dysmenorrhea for 6 months and malodorous vaginal discharge for 1 month	NA	IIA	Hysterectomy, bilateral salpingo-oophorectomy, and bilateral LN dissection	nil	Not involved	NA	NA
Tiltman, A. J. [23]	1982	3	Continuous vaginal bleeding for 6 months	NA	IIIB	#171717; Radiation therapy	nil	NA	6	Expired due to uremia
			Intermittent intermenstrual vaginal bleeding for 4 months	NA	#171717; IIIB	#171717; Radiation therapy	nil	NA	43	No signs of recurrence
			NA	NA	NA	Radical hysterectomy with pelvic lymphadenectomy	nil	NA	24	No signs of recurrence
Kashimura, M. [24]	1984	1	Vaginal discharge	NA	IB	Total abdominal hysterectomy and partial vaginectomy	nil	NA	48	No signs of recurrence
Maeyama, M. [25]	1985	1	NA	Not detected	IIA2	Hysterectomy, and bilateral salpingo- oophorectomy	nil	NA	10	No signs of recurrence
Degefu, S. [26]	1986	1#	Weight loss	NA	IIB (clinical)	Radiation therapy	nil	NA	15	Expired of progressive inanition and uremia
Wong, W. S. [14]	1990	1	NA	Not detected	IIA (clinical)	Hysterectomy	nil	NA	49	No signs of recurrence
de Jesus, M [27]	1990	1	Yellowish vaginal discharge for 4 months	Not detected	IB3	Radical hysterectomy with pelvic lymphadenectomy	nil	Not involved	24	No signs of recurrence
Pantanowitz, L. [28]	2003	1	Vaginal bleeding	Not detected	IIA2	Radical hysterectomy with bilateral pelvic lymphadenectomy	nil	Not involved	NA	NA
Yorganci, A. [29]	2003	1ˆ	Postmenopausal vaginal bleeding for 1.5 months	Detected	NA	Type II radical hysterectomy with bilateral salphingo- oopherectomy and total vaginectomy	nil	NA	20	Recurrence at the introitus
Frega, A. [4]	2007	3	NA	High risk HPV types	IB	Radical hysterectomy, bilateral salphingo-oophorectomy, upper vaginal third colpectomy and pelvic lymphadenectomy	nil	Not involved	55 (mean)	No signs of recurrence
Anghel, R. M. [10]	2017	1	Genital hemorrhage, pelvic pain, and dyspareunia for 6 months	NA	IIIB	Radical hysterectomy	Radiation therapy	NA	3	Recurrence at bladder, rectum, and appendix

NA: not available. LN: lymph node.

∗Patient previously had subtotal hysterectomy and bilateral salpingo-oophorectomy.

#The other case had a metastasis of the lung; thus, we did not discuss here.

ˆA case with vaginal and concurrent cervical VCs

Based on current literature reviews, most patients presented with abnormal vaginal bleeding. Hence, for those with chronic, recurrent or massive vaginal bleeding, speculum examination should be performed to exclude vaginal or cervical lesions. If suspicious of malignant lesion exists, extensive cervical biopsy is essential for further pathologic diagnosis. Repeated biopsy may be considered for patients with initial negative pathologic reports and high clinical suspicions of malignancy.

Most cases from Table 1 did not report the HPV status. Among the eight cases whose HPV statuses were available, four of them were not-detected. The other four cases demonstrated high-risk HPV typing, and this result might implicate the association of HPV and VC development [4]. However, unlike the confirmatory association between cervical invasive SCC and HPV infection, the relationship between VC and infestation of HPV is still under debate. When it comes to the VC of the vulva, previous research showed a low detection rate of HPV infection and this also raised the question of the causality between them [13].

With regard to management, wide local excision or simple hysterectomy are recommended as the first-line therapeutic approaches for cervical VC [5]. Accurate diagnosis of VC is essential because it prevents the patients from undergoing unnecessary radical surgery and being exposed to harmful radiation [14]. As Table 1 shows, surgical management was performed in nineteen cases (70.4%, 19/27) as primary treatment. After excluding two cases whose outcomes are not available, twelve of the surgical cases (70.6%, 12/17) showed no recurrence. With regard to irradiation, eight cases received radiation therapy as the primary treatment. One was expired due to uremia after radiation therapy within 6 months and was excluded. Five of them (71.4%, 5/7) revealed no recurrence. Although previous study showed that radiation therapy was prohibited due to the possibility of anaplastic transformation [8], our review did not demonstrate significantly differences of recurrence betweensurgery and radiation therapy. We still recommend surgical management as first-line therapyas most authors agreed. If the patient is contraindicated for surgery, irradiation may be an alternative treatment with comprehensive share-decision-making addressed before radiation therapy, and regular follow-up for distant metastasis should be considered.

As mentioned, the possibility of regional lymphatic involvement was low although there was a highly avid uptake of FDG and enlarged left pelvic lymph node in our case. In the previous published cases of cervical VC, all removed lymph nodes were free of malignancy [5, 7, 15]. Thus, routine lymph node dissection has no consensus in the treatment of VC underwent surgical intervention [7].

By reviewing the historical cases listed in Table 1, we concluded that most patients with VC had abnormal vaginal bleeding as the initial manifestation. This finding aligned with our patient. Besides, the stages of the most reported cases ranged from FIGO staging IB to IIA. Similarly, as aforementioned, the pathologic stage of our case was IIA. In Table 1, we found that 70.4 % of reported cases received surgical excision as the primary treatment, and 70.6% of them showed no recurrence during the follow-up period. In most cases, adjuvant therapy was not applied. Back to our case, we performed surgical therapy as the primary management. With regular follow-up at outpatient department, no recurrence was found for two year, and this relatively favorable outcome was similar to other historical cases. One of the inconclusive issues is that the prevalence of HPV detection in VC. In Table 1, eight cases received HPV investigation, only half of them had high risk HPV.. Our patients belong to the HPV-independent group. The final pathological report of our case contradicted the presence of presumably lymph node metastasis, which was consistent with prior studies [5, 7, 15]. On the other hand, the “false positive” nature of MRI based solely on the diameter of a retroperitoneal lymph node was well established in the literature. However, the false positivity under the imaging of PET/CT with high SUV (both early and late phases) is an issue of needed further discussion.

## 4 Conclusion

VC is difficult to diagnose preoperatively. We suggest extensivecervical biopsy with sufficient stroma depth of exophytic and endophytic growth pattern as an initial diagnosis. Imaging studies such as PET/CT, CT or MRI are alternative in cases of undetermined nature. with hysterectomy planned in suspicious of clinical malignancy.

## Declarations

### Author contribution statement

All authors listed have significantly contributed to the investigation, development, and writing of this article.

### Funding statement

This research did not receive any specific grant from funding agencies in the public, commercial, or not-for-profit sectors.

### Data availability statement

Data will be made available on request.

### Declaration of interest’s statement

The authors declare no conflict of interest.

### Additional information

The authors have informed the patient through phone call about the written of this case in the form of case report and having the verbal approval from the patient personally on July 2, 2021.
